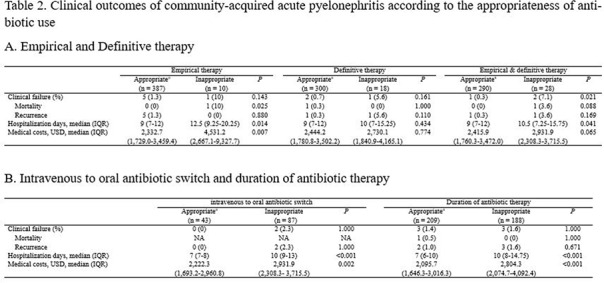# Differences in the Clinical Outcome of Community-Acquired APN According to the Appropriateness of Antibiotic Use

**DOI:** 10.1017/ash.2021.12

**Published:** 2021-07-29

**Authors:** Bongyoung Kim, Choseok Yoon, Se Yoon Park, Ki Tae Kwon, Seong-yeol Ryu, Seong-Heon Wie, Hyun-uk Jo, Jieun Kim, Kyung-Wook Hong, Hye In Kim, Hyun ah Kim, Mi-Hee Kim, Mi-Hyun Bae, Yong-Hak Sohn, Jieun Kim, Yangsoon Lee, Hyunjoo Pai

## Abstract

**Background:** The purpose of this study was to find out the relationship between appropriateness of antibiotic prescription and clinical outcomes in patients with community-acquired acute pyelonephritis (CA-APN). **Methods:** A multicenter prospective cohort study was performed in 8 Korean hospitals from September 2017 to August 2018. All hospitalized patients aged ≥19 years diagnosed with CA-APN at admission were recruited. Pregnant women and patients with insufficient data were excluded. In addition, patients with prolonged hospitalization due to medical problems that were not associated with APN treatment were excluded. The appropriateness of empirical and definitive antibiotics was divided into “optimal,” “suboptimal,” and “inappropriate,” and optimal and suboptimal were regarded as appropriate antibiotic use. The standard for the classification of empirical antibiotics was defined reflecting the Korean national guideline for the antibiotic use in urinary tract infection 2018. The standards for the classification of definitive antibiotics were defined according to the result of in vitro susceptibility tests of causative organisms. Clinical outcomes including clinical failure (mortality or recurrence) rate, hospitalization days, and medical costs were compared between patients who were prescribed antibiotics appropriately and those who were prescribed them inappropriately. **Results:** In total, 397 and 318 patients were eligible for the analysis of the appropriateness of empirical and definitive antibiotics, respectively. Of these, 10 (2.5%) and 18 (5.7%) were inappropriately prescribed empirical and definitive antibiotics, respectively, and 28 (8.8%) were prescribed either empirical or definitive antibiotics inappropriately. Patients who were prescribed empirical antibiotics appropriately showed a lower mortality rate (0 vs 10%; *P* = .025), shorter hospitalization days (9 vs 12.5 days; *P* = .014), and lower medical costs (US$2,333 vs US$4,531; *P* = .007) compared to those who were prescribed empirical antibiotics “inappropriately.” In comparison, we detected no significant differences in clinical outcomes between patients who were prescribed definitive antibiotics appropriately and those who were prescribed definitive antibiotics inappropriately. Patients who were prescribed both empirical and definitive antibiotics appropriately showed a lower clinical failure rate (0.3 vs 7.1%; *P* = .021) and shorter hospitalization days (9 vs 10.5 days; *P* = .041) compared to those who were prescribed either empirical or definitive antibiotics inappropriately. **Conclusions:** Appropriate use of antibiotics leads patients with CA-APN to better clinical outcomes including fewer hospitalization days and lower medical costs.

**Funding:** No

**Disclosures:** None

**Table 1.**
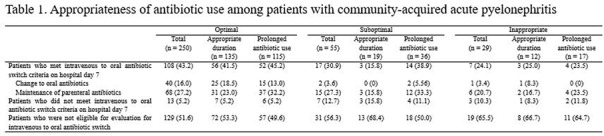

**Table 2.**